# The Role of Macrophages in the Pathogenesis of SARS-CoV-2-Associated Acute Respiratory Distress Syndrome

**DOI:** 10.3389/fimmu.2021.682871

**Published:** 2021-05-10

**Authors:** Anna Kosyreva, Dzhuliia Dzhalilova, Anastasia Lokhonina, Polina Vishnyakova, Timur Fatkhudinov

**Affiliations:** ^1^ Department of Neuromorphology, Science Research Institute of Human Morphology, Moscow, Russia; ^2^ Histology Department, Peoples Friendship University of Russia (RUDN University), Moscow, Russia; ^3^ Department of Immunomorphology of Inflammation, Science Research Institute of Human Morphology, Moscow, Russia; ^4^ Department of Regenerative Medicine, National Medical Research Center for Obstetrics, Gynecology and Perinatology Named After Academician V.I. Kulakov of Ministry of Healthcare of Russian Federation, Moscow, Russia; ^5^ Department of Growth and Development, Science Research Institute of Human Morphology, Moscow, Russia

**Keywords:** M1/M2 macrophages, inflammation, ARDS, cells therapy, SARS-CoV-2

## Abstract

Macrophages are cells that mediate both innate and adaptive immunity reactions, playing a major role in both physiological and pathological processes. Systemic SARS-CoV-2-associated complications include acute respiratory distress syndrome (ARDS), disseminated intravascular coagulation syndrome, edema, and pneumonia. These are predominantly effects of massive macrophage activation that collectively can be defined as macrophage activation syndrome. In this review we focus on the role of macrophages in COVID-19, as pathogenesis of the new coronavirus infection, especially in cases complicated by ARDS, largely depends on macrophage phenotypes and functionalities. We describe participation of monocytes, monocyte-derived and resident lung macrophages in SARS-CoV-2-associated ARDS and discuss possible utility of cell therapies for its treatment, notably the use of reprogrammed macrophages with stable pro- or anti-inflammatory phenotypes.

## Introduction

Since its initial outbreak in Wuhan (China) in 2019, the novel coronavirus infection has rapidly progressed into pandemic. The situation required massive counter measures with distinct signs of global emergency and drastic restrictions in human activities. Under the lack of efficient antivirals, fatality rates for the coronavirus disease of 2019 (COVID-19) are disastrously high. The main efforts in COVID-19 patient management are aimed at symptomatic and pathogenetic treatment, and effective new methods of dealing with the consequences are in great demand. In severe cases of COVID-19, the virus promotes cytokine storm with uncontrolled massive release of pro-inflammatory cytokines leading to acute respiratory distress syndrome (ARDS) and acute heart failure; these conditions are highly life-threatening and fraught with acquisition of secondary bacterial infections. Prevention of the cytokine storm in SARS-CoV-2-infected individuals remains extremely relevant, and safe means of such prevention stay among primary goals of medical research worldwide. As demonstrated, ARDS can be safely managed by cell therapy including the use of mesenchymal stromal cells (MSCs); the option can be enhanced by *in vitro* reprogramming of MSCs into anti-inflammatory macrophages (M2 macrophages) prior to administration. This review elucidates the role of macrophages in ARDS pathogenesis with a special focus on SARS-CoV-2-associated ARDS (COVID-19-ARDS). Identification of cellular and molecular mechanisms related to the role of macrophages in ARDS is highly relevant for the development of potential treatment strategies for COVID-19.

Severe acute respiratory syndrome-related coronavirus 2 (SARS-CoV-2) belongs to *Coronaviridae* family. Its particles contain single-stranded (+) RNA enclosed within a complex assembly of proteins responsible for entering a host cell and viral replication, including the membrane (M), envelope (E), nucleocapsid (N), and spike (S) proteins (1); S contains a receptor-binding domain that specifically recognizes angiotensin-converting enzyme 2 (ACE2) as its receptor; that is essential for penetration of the virus into alveolar epithelial cells (pneumocytes) type II which express ACE2 (2, 3). The interaction of S protein with ACE2 in lung epithelium promotes local enforcement of angiotensin II production and increase in bradykinin levels, leading to a surge of inflammatory response (4).

Moreover, the binding of SARS-CoV-2 S protein to ACE2 promotes formation of multinucleated syncytia (4, 5). S protein at the surface of infected cells effectively promotes their fusion with neighboring cells; the resulting syncytia may incorporate epithelial and myeloid cells (6). Although formation of syncytia in the lungs of patients with SARS-CoV-2 has been confirmed, its role in viral persistence and dissemination needs to be specified.

Clinical manifestations of COVID-19 begin on day 2–14 after a contact with infected carrier (day 5 on average) (7). The infection may lead to lethal outcome on day 6–41 after the first symptoms (day 14 on average). Duration, severity and lethality of the disease depend on the patient’s age and immune status (8, 9), the presence of chronic comorbidities (notably diabetes mellitus, chronic hypertension with vascular failure, chronic inflammatory conditions of the lungs, and malignant neoplasms) (10), genetic features, obesity status, and glucose levels (11, 12). For instance, in children with the age-related physiologically strong innate immunity clinical symptoms of COVID-19 are less pronounced than in adults (10). Severe obesity (BMI ≥ 35 kg/m^2^) has been associated with intensive care unit admission, whereas histories of heart disease and obesity (BMI ≥ 30 kg/m^2^) have been independently associated with the use of invasive mechanical ventilation (11).

Clinical manifestations of COVID-19 vary dramatically from asymptomatic carriage to acute respiratory distress syndrome; typical symptoms include fever, dry cough, breath shortness, myalgia, and fatigue (8, 13, 14). Severe coronavirus-associated pneumonia results from rapid replication of the virus in epithelial cells with their death accompanied by massive infiltration of macrophages and neutrophils to intra-alveolar septa and alveolar lumina. Extremely high production of pro-inflammatory mediators by the infiltrating immune cells leads to acute lung injury (ALI) and ARDS (15, 16). The pronounced inflammatory response in coronavirus infections can play both protective and destructive roles (15–17). Activation of immune cells provides elimination of the viruses but its excessive infiltration and functioning can lead to tissues injury. Massive release of pro-inflammatory mediators by infiltrating immune cells combined a direct cytotoxic effect of the virus on epithelial cells result in lung injury in SARS-CoV-infected patients (18).

SARS-CoV-2 is similar to other coronaviruses, such as SARS-CoV-1 and MERS-CoV (19, 20). Similarly with the fatal cases of SARS-CoV-1 and MERS-CoV infections, lung autopsies of patients who died of COVID-19 reveal extensive cellular infiltration with the predominance of macrophages. High levels of IFNγ, IL-6, IL-12, TGFβ, CCL2, CXCL9, CXCL10 and IL-8 are typical for both SARS-CoV-2- and SARS-CoV-1-associated fatal cases, with higher levels of IL-1β and lower levels of IL-10 for SARS-CoV-1.

For SARS and MERS coronaviruses, severity of the respiratory syndrome positively correlates with macrophage numbers (21, 22). Viral proteins obtained from SARS-CoV-1 stimulate the release of proinflammatory cytokines by peripheral blood monocytes (23). Macrophages and monocytes of peripheral blood can be activated by SARS-CoV-1 and recruit neutrophils and immune effectors (NK-, T-, and B-cells) to form early adaptive immunity (24, 25).

Given the important role of macrophages in SARS-CoV-2-induced immune responses, targeted modulation of their functional activity may provide a new approach for the management of patients with severe COVID-19.

## Acute Respiratory Distress Syndrome (ARDS)

Acute lung injury (ALI) rapidly progressing into ARDS is the major cause of high fatality in pneumonias, including SARS-CoV-2-associated pneumonia (2, 26, 27). Transition of ALI into ARDS results from diffuse alveolar damage (28), usually a consequence of excessive and uncontrolled systemic inflammatory reactions in response to lung injury caused by trauma, burn, pneumonia, sepsis, etc., violating endothelial and epithelial barriers (29). COVID-19-associated patterns of diffuse alveolar damage are typical for ARDS (30–32). ARDS develops in 42% of COVID-19 patients with pneumonia, or in 61–81% of intensive care patients with COVID-19 (33). The overwhelming prevalence of ARDS (90%) among fatal complications of COVID-19 was demonstrated on a sample of 2000 autopsies; other fatal complications included pulmonary embolism (6%) and sepsis (1.5%) (31). It should be noted that the prognosis for SARS-CoV-2-associated ARDS (COVID-19-ARDS) is worse than for ARDS of other genesis. In-hospital mortality for ARDS in general does not exceed 35.3% (33); for COVID-19-ARDS, it has been estimated 62% and reaches 65.7–94% for intensive care patients on ventilators. Risk factors for unfavorable prognosis include old age, the presence of comorbidities (hypertension, cardiovascular diseases, diabetes, renal failure), as well as reduced lymphocyte counts and elevated levels of D-dimer. Death from COVID-19-ARDS occurs mostly from respiratory failure either by itself (53%) or accompanied by cardiac failure (33%), and much less frequently from myocardial injury and/or circulatory failure (7%) (33).

At the initial stages of ALI, immune response is triggered by activation of antigen-presenting cells, including macrophages and dendritic cells, which facilitates production of pro-inflammatory cytokines (notably TNFα, IL-1, and IL-6), as well as prostaglandins and histamine (34, 35). These substances boost endothelial permeability and facilitate homing/migration of neutrophils, macrophages, and lymphocytes to the focus of inflammation, which exacerbates the injury and promotes ARDS. Accumulation of neutrophils in blood capillaries and inter-alveolar septa leads to intra-alveolar edema and avalanche ROS production which contribute to hypoxemia and critical deterioration of lung function (36). The increased permeability of microcirculatory vessels obliterates the air-blood barrier while promoting hemorrhages and fibrin deposition (37).

Analysis of the aforementioned sample of 2000 autopsies revealed similar pathological alterations in the affected lung tissues, differing only by the degree of expansion. These alterations can be described as diffuse alveolar damage with underlying microcirculatory failure (microangiopathy, thrombosis, and occasionally the destructive-productive vasculitis) and pulmonary hemorrhage. Such interstitial viral pneumonia with a pronounced vascular and hemorrhagic component represents the invariant morphological substrate of COVID-19-ARDS (31).

ARDS pathogenesis proceeds in several phases, most commonly defined as exudative phase, proliferative (rehabilitation) phase, and fibrotic (fibroproliferative) phase (38–40).

Exudative phase of ARDS is distinguished by diffuse alveolar damage with massive death of epithelial and endothelial cells. COVID-19 predominantly affects the respiratory system with only minor damage to other organs; it is assumed that SARS-CoV-2 has a negative effect on alveolar epithelial cells (pneumocytes) and endothelial cells (41). The massive death of pneumocytes severely violates gas exchange (42); concomitant damage to endothelium with the resulting increase in capillary permeability lead to perivascular and intra-alveolar edema with transudation of plasma proteins, notably fibrinogen B, to the alveoli. Early stages of ARDS pathogenesis are marked with massive activation and immigration of circulating neutrophils (43, 44). Edema-associated damage to pneumocytes type II results in decreased production of surfactant and increased production of inflammation mediators (e.g. pro-inflammatory cytokines IL-1β, IL-6, and IL-18) which stimulate homing of macrophages and neutrophils to the lungs by chemotaxis (45, 46). Endothelial dysfunction and disrupted oxygenation of pulmonary tissues leads to avalanche ROS production (47). Bacterial invasion, which is possible already in this phase, may lead to development of bacterial pneumonia and sepsis. Formation of hyaline membranes in the alveoli (indicative of ARDS) proceeds throughout this phase (5); the presence of multinucleated giant cells comprising markers of both macrophages and pneumocytes (48) indicates the scavenging involvement of macrophages.

Exudative phase of ARDS ends with the transition to proliferative (rehabilitation) phase marked by facile regeneration of pneumocytes and endothelium under conditions of suppressed pro-inflammatory cytokine production (49, 50). As neutrophils die massively by efferocytosis, macrophages accomplish debridement of the site by phagocytosis and their capacity for production of anti-inflammatory factors (e.g. IL-10 and TGF-β) increases (50). Phagocytosis is competitive with iNOS expression and stimulates Arg-1 expression thus inhibiting ROS production by macrophages (49).

The processes of prolonged recovery of lung tissue after infection are also considered a phase of ARDS, as 30–50% of adult patients with ARDS develop a full-scale inflammatory response leading to profound histoarchitectural alterations, development of fibrosing alveolitis with cystic restructuring, and overall deterioration of pulmonary function accompanied by abnormal activation of macrophages, fibrocytes, and fibroblasts (29, 51). Signs of pulmonary fibrosis can be clearly detected by computer tomography in about 17% of patients with confirmed COVID-19; additional fibrotic lesions with gradual replacement of epithelial cells by collagen and extracellular matrix (ECM) components can be formed during the post-pneumonia recovery (52, 53). At present, the true relationship between pronounced pulmonary fibrosis and prognosis in COVID-19 patients is debatable. Some evidence (52) associates the presence of fibrosis with favorable prognosis and stabilization of the patient’s condition. Nevertheless, the outcome of the inflammatory process certainly depends on the prevalence and progression of pulmonary fibrosis (54, 55).

Pulmonary fibrosis (sometimes marked as a delayed complication of ARDS, not a fibrotic phase of ARDS itself) results from the excessive proliferation of fibroblasts and excessive production of ECM components by these fibroblasts (39, 56). At this stage, patients need long-term respiratory support by mechanical ventilation, and the mortality rates are extremely high (56). At the tissue level, this phase is marked by release of various pro-fibrotic molecules (e.g. TGF-α, TGF-β, IL-1β, and the platelet-derived growth factor) which promote excessive deposition of fibronectin, collagens, and other ECM components (57). The fibro-proliferative response is facilitated by elevated expression of pro-angiogenic cytokines and growth factors by immune cells (e.g. MIP-2, angiopoietin 2, and VEGF) (58–61). Mechanical ventilation may exacerbate fibrosis progression by damaging the epithelium and interfering with surfactant production, ultimately promoting the ventilator-associated lung injury (62–65).

As macrophage participation is essential for every phase of ARDS development, these cells represent a potential target/tool for ARDS therapy.

## General Characterization of Macrophages

Macrophages are cells mediating both innate and adaptive immunity; they play major roles in various physiological and pathological processes, and can be involved in tissue injury as either injury-inducing or repair-promoting. Exemplary recruited macrophage precursors, blood monocytes, originate in the bone marrow and constitute about 5–9% of peripheral blood leukocytes. Monocytes reside in circulation for 1–2 days; by the end of that period, monocytes either die by apoptosis or relocate from circulation into tissues where they further differentiate into macrophages (66). Resident macrophages such as alveolar macrophages (AMs), Kupffer cells, microglial cells, Langerhans cell, macrophages of splenic red pulp and peritoneal macrophages originate prenatally from the yolk sac and embryonic liver (67, 68).

In healthy humans, blood monocytes comprise three subpopulations including classical (CD14++CD16−, about 90%), non-classical (CD14+CD16++, about 5–10%), and intermediate monocytes (CD14++CD16+, the smallest subpopulation of 1–5%) (69). These subpopulations are distinguished by expression levels of chemokine receptors, as well as by phagocytic capacity and distribution in particular tissues under physiological conditions and/or in the course of inflammatory processes. CD14++ monocytes are considered mature; they have pronounced phagocytic activity and can produce reactive oxygen species (ROS) and cytokines upon Toll-like receptor (TLR) activation (70). CD16++ cells are engaged in synthesis of cytokines (predominantly pro-inflammatory) and do not produce ROS (69, 70). The role of intermediate subpopulation is unclear; given the high expression level of MHC-II on these monocytes, they are suggested involved in antigen presentation and T cell activation (71).

Some additional monocyte phenotypes have been characterized in recent studies based on gene and protein expression patterns (72–74). Under pathological conditions, the ratio between different monocyte subpopulations may be shifted; increased severity of inflammatory diseases is usually associated with increased proportions of non-classical and intermediate (CD16+) monocytes (75, 76).

Under physiological conditions, a considerable proportion of circulating monocytes spontaneously (under weak chemokine signaling) migrate into tissues and differentiate into resident macrophages which implement homeostatic and regulatory functions by scavenging dead cells and carrying out immune surveillance (77, 78). Under inflammatory conditions, monocytes become strongly attracted to the focus of inflammation where they differentiate into pro-inflammatory macrophages with enormously enhanced phagocytic and bactericidal activity. In comparison with the resident macrophages that survive in tissues for years, these pro-inflammatory macrophages are transient (survive for few weeks at most).

**Figure 1:**
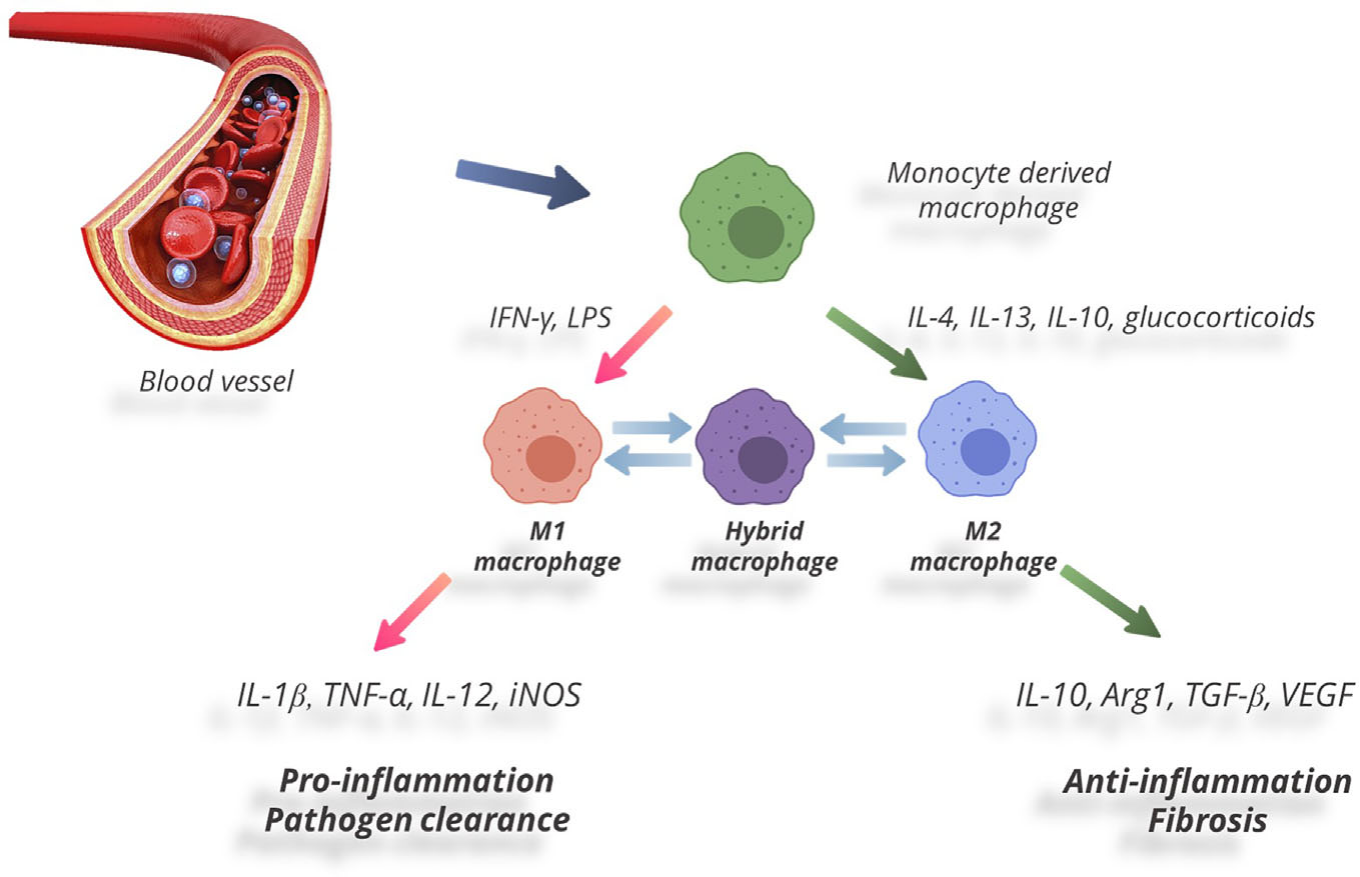
Two functional subtypes of macrophages.

As a cell type, macrophages show high plasticity, i.e. they are capable of switching phenotypes (79, 80). The process of switching phenotypes and developing specific reactions to microenvironment by macrophages is called ‘polarization’. One of important factors directing macrophage polarization is local cytokine levels. Peripheral tissues in mammals (including humans, mice, and rats) were shown to comprise two functional subtypes of macrophages (**Figure 1**): the ‘classically’ activated pro-inflammatory macrophages (commonly designated M1) and the ‘alternatively’ activated anti-inflammatory macrophages (commonly designated M2); the distinction has been based on expression of specific markers and production of biologically active substances (81–86). M1 polarization is usually a response to pro-inflammatory cytokines, e.g. IFN-γ and TNFα, as well as pathogen-associated stimuli, e.g. lipopolysaccharide (LPS). M1 macrophages produce TNFα, IL-1, IL-6, IL-12, and IL-23, as well as chemokine CCL8, monocyte chemotactic protein 1 (MCP-1), macrophage inflammatory protein 2 (MIP-2), ROS, cyclooxygenase 2 (COX-2), CD16, and CD32. Expression of inducible NO-synthase (iNOS) by macrophages is a marker of M1 polarization in rodents. M1 macrophages are engaged in developing pro-inflammatory reactions, chemotaxis, production of free radicals, degradation of extracellular matrix, and antimicrobial and antitumor activities (38, 87).

The opposite type of macrophage polarization, M2, occurs in response to Th2 cytokines (IL-4, IL-13) and anti-inflammatory cytokines — notably IL-10 and transforming growth factor beta (TGF-β) (88). M2 macrophages are distinguished by high expression levels of IL-1 receptor antagonist (IL-1RA), arginase 1 (Arg-1), IL-10, TGF-β, CCL18, and low production of IL-12. M2 macrophages reduce inflammation by suppressing effector T cells; they promote wound healing and tissue repair by producing growth factors (VEGF, EGF, PDGF, IL-10). They also participate in Th2-mediated reactions and extracellular matrix remodeling (88–92). Under pathological conditions a shift in M1/M2 balance towards the anti-inflammatory М2 phenotype has been shown to facilitate regeneration and repair. Positive effects of M2 macrophages were demonstrated under conditions of injury, including skin wound healing in mice with diabetes (93), experimental spinal damage (94–96), cardiomyopathy in mice (97), prevention of atherosclerotic lesions in experimental models (98), and management of myocardial infarction in patients with diabetes (99, 100).

The M1/M2 paradigm has been lately criticized as too primitive. Recent data demonstrate that macrophages tend to present with a continuum of phenotypes even in the absence of inducing stimuli (101). The continuous nature of macrophage plasticity manifests itself under various physiological and pathological conditions (102). The switching between pro- and anti-inflammatory states has been reviewed in depth elsewhere (102, 103), with states of indistinct functionality lacking clear M1 or M2 markers conventionally classified as hybrid phenotypes (102, 104) (**Figure 1**). Despite the widespread opinion that the M1/M2 paradigm reflects *in vitro* extremization of the *in vivo* conditions, it still provides a good framework for many experimental findings (105–107).

As the major cytokine-producing and antigen-presenting cells, macrophages control effector T-cell homeostasis, promote T-cell priming, and induce Th17 cell differentiation (108). In total, macrophages mediate innate immune responses directly and make a crucial contribution to the effector phase of adaptive immune response (109). Infection of macrophages by SARS-CoV-2 impairs adaptive immune responses against the virus (110).

## Lung Macrophages

Two distinct populations of lung macrophages (LMs) are both of mixed origin, derived prenatally from the yolk sac and fetal liver and postnatally from the bone marrow (111, 112). The first and most abundant of them, alveolar macrophages (AMs), is associated with the epithelial walls of alveoli (113).

AMs express peroxisome proliferator-activated receptor gamma (PPARγ, a regulator of lipid metabolism) under the action of granulocyte-macrophage colony-stimulating factor (GM-CSF) (114); their main function is phagocytosis and surfactant catabolism (115). AMs are further divided into two subpopulations by their functional state and sources of origin: long-lived resident AMs and recruited AMs (39). The long-lived resident AMs are uniform, quiescent, and immunosuppressive; they mainly present with M2 phenotypes (39, 116). Resident AMs are sentinels at the tissue/air interface; they implement immune surveillance and maintain tissue homeostasis (38, 116, 117). They are counterbalanced by peripheral blood monocytes recruited from circulation to the alveoli in response to certain stimuli (39); these monocytes differentiate into ‘recruited’ M1 AMs (117). Accumulating evidence suggests that both resident and recruited AMs are deeply involved in ARDS (39, 40).

The second population comprises interstitial macrophages (IMs), which account for 30–40% of LMs (113). In contrast to AMs, which differentiate from circulating monocytes during inflammation only (118), IMs can be classified as resident monocyte-derived macrophages (113). IMs have been associated with tissue remodeling and repair, antigen presentation, and modulation of dendritic cell functions (113, 119).

Recently, a new IM subpopulation of Nerve- and Airway-associated Macrophages (NAMs) has been characterized in mice (120). NAMs are distinct from other lung-resident macrophage subsets; they express higher levels of MHCII, Cx3cr1, C1q, C3ar1, and F13a1 and lower levels of Lyve1 (Lymphatic vessel endothelial hyaluronan receptor 1) than other macrophage populations (120). Involved in maintenance of immune and tissue homeostasis, NAMs also regulate infection-induced inflammation through secretion of immunosuppressive factors such as IL-10.

A minor population of LMs is constituted by intravascular macrophages (111). As shown by Evren et al. (121), lung vasculature contains not only patrolling CD14loCD16+ and extravasating CD14+ monocytes but also intravascular macrophages. Despite populating the same anatomical niche, intravascular macrophages are distinct from blood monocytes in terms of morphology, phenotype, and gene expression. Intravascular LMs are functionally related to red pulp macrophages of the spleen and Kupffer cells of the liver; they preferentially express genes involved in red blood cell turnover and iron metabolism (CD163, HMOX1, FTL, SLC40A1, SLC48A1) and genes related to leukocyte-endothelium adhesion (121).

As other tissue macrophages both AMs and IMs can be divided into two functional phenotypes, that is microenvironment-depended: M1 macrophage and M2 macrophage (122, 123). Mitsi et al. (124) demonstrated that resident AMs have mostly hybrid phenotypes, i.e. express M1 and M2 markers simultaneously, and that such hybrid phenotypes of resident AMs are stable in human populations independently of geographic habitat. Specht at al (101). used single-cell proteomic analysis to demonstrate co-expression of M1 and M2 signatures in cultured AMs in the absence of polarizing stimuli. The existence of stable hybrid M1/M2 phenotypes is clearly beyond the conventional (original) views on macrophage polarization. Such hybrid phenotypes may enable rapid adaptation of resident AMs to the demands of their volatile microenvironment by switching between M1-and M2-specific functions in response to particular stimuli.

## Macrophage and Monocyte Functionalities in Viral Infections

Macrophages and monocytes are the first immune cells to interact with viral pathogens; they can be infected and may become instrumental for spreading of the virus (125). They can contribute to virus dissemination by interaction with other populations of cells through direct cell-to-cell contacts. Macrophages and monocytes actively migrate to the sites of damage while producing chemokines and cytokines that play important roles in immunity reactions and inflammatory processes. Such behaviors can be employed by viruses for spreading throughout the body to various tissues and organs including the brain. The penetration of infected monocytes through blood-tissue barriers, notably the ‘Trojan horse’ delivery of viral particles into the central nervous system, is common for human immunodeficiency virus (HIV) (126–128), human herpes virus (HCV) (129), human cytomegalovirus (HCMV) (125) and Japanese encephalitis virus (JEV) (130) infections. It provides stable viral pools capable of sudden reinforcement upon optimal conditions for their reactivation.

Viruses have to come over the particular barriers to interact with macrophage or monocyte in order to infect and replicate in these cells. Based on inner genetic program, in three days of circulation in blood flow, monocytes have to either follow the apoptosis pathway or differentiate into tissue macrophages (125). In such case, viruses demonstrate their ultimate potential to modulate and moderate cells to avoid degradation and instead replicate in the organism effectively. They decline apoptosis route and prolong cell’s life span thanks to apoptotic pathways regulation (NFkB and PI3K), which suggest microRNAs involvement (131, 132) and the mitochondrial pathway modulation (133). They provide cell polarization changes, disguise viral receptors from cell surface and effect on cytokine/chemokine expression in order to elude from the immune response. They also provide the influence on cells motility in order to make the viral distribution through the body. This can facilitate the disorders, chronic inflammation and further predisposition to the cognitive pathologies, multiple sclerosis, inflammatory demyelinating disease, precancerous conditions and cancer (134).

Although macrophages and monocytes of peripheral blood are capable of delivering viruses to organs and tissues (135), it has been shown that these cells are infected by SARS-CoV-1 non-productively, i.e. the virion RNA is synthesized, but the virus is produced non- or almost non-infectious (136). Nevertheless, the macrophage/monocyte responses are central to SARS pathogenesis (137, 138). It should be noted that, while SARS-CoV-1 infection in mononuclear phagocytes is abortive, MERS-CoV can replicate in monocytes, macrophages, and dendritic cells (16).

## The Role of Monocytes and Monocyte-Derived Macrophages in SARS-CoV-2-Associated ARDS

Abrupt and rapidly progressing clinical manifestations of COVID-19, peaking at 7–10 days after the onset, have been identified with the long-known phenomenon of cytokine storm (139). The systemic reaction, which results from massive macrophage activation and has been accordingly designated ‘macrophage activation syndrome’ (MAS), plays the central role in SARS-CoV-2-associated complications including ARDS, disseminated intravascular coagulation syndrome (DICS), edema, and pneumonia (2, 26, 27, 140). As demonstrated by Huang et al. (141), COVID-19 patients (notably those admitted to intensive care units) have elevated blood levels of IL-1β, IL-2, IL-7, IL-9, IL-10, IL-17, G-CSF, GM-CSF, IFN-γ, TNF-α, CXCL8, CXCL10, MCP1, MIP1A, and MIP1B. Although blood counts of monocytes/macrophages in COVID-19 patients often stay within the reference range, proportion of activated cells among them is dramatically elevated.

Although studies devoted to the role of macrophages in COVID-19 are few, considerable efforts have been focused on phenotypes and counts of blood monocytes. Zhou et al. (142) studied a cohort of 33 hospitalized patients diagnosed with COVID-19 and observed that the counts of CD16+ monocytes (including non-classical CD14+CD16++ and intermediate pro-inflammatory CD14++CD16+ subpopulations with high expression of IL-6) were significantly increased, especially in patients who developed ARDS. Specifically, relative counts of intermediate monocytes in patients with severe COVID-19 were increased to 45% of all blood monocytes (compared with 1–5% in healthy individuals). This finding highlights the important role of monocytes in generalization of the inflammatory reactions inflicted by the new coronavirus infection.

Zhang et al. (143) performed a detailed study of expression profiles for the intermediate pro-inflammatory CD14++CD16+ subpopulation of blood monocytes in a cohort of 28 COVID-19 patients. The authors observed expression of both pro-inflammatory (CD80+) and anti-inflammatory (CD163+, CD206+) differentiation markers by these cells, as well as their positivity for pan-macrophage marker CD68+. These results indicate that, under given conditions of acute progression of the disease, monocytes differentiate into macrophages while still in the circulation. This newly identified population of circulating monocytes/macrophages produces IL-6, TNFα, and IL-10 at high levels, which could effectively promote the cytokine storm. The counts of such cells were the highest in hospitalized patients with severe COVID-19 and positively correlated with the length of stay in intensive care units. Consistently with other studies, the counts of non-classical and intermediate monocytes in COVID-19 patients were heavily increased, against a decrease in the classical monocyte counts (143). In monocytes, SARS-CoV-2 stimulated the default transcriptional program (enriched with M2-type genes), as CD163 expression in SARS-CoV-2-infected monocytes was significantly higher, whereas HLA-DR expression was decreased, as compared with non-infected controls (144).

As stated before, viral particles of SARS-CoV-2 provide no productive infection in mononuclear phagocytes (144). Moreover, Liu et al. (145) revealed no significant differences in surface expression of CD80, CD86, CD72, and PD-L1 on monocytes between COVID-19-convalescent individuals and matched SARS-CoV-2-unexposed individuals, indicating the lack of long-term impact of SARS-CoV-2 on monocytes (145). However, another study demonstrates that SARS-CoV2 triggers mitochondrial ROS production in monocytes, which induces stabilization of hypoxia-inducible factor-1α (HIF-1α) and consequently promotes glycolysis (12). The elevated glucose levels enhance SARS-CoV-2 replication in monocytes and induce cytokine expression, thus directly inhibiting T cell response and reducing epithelial cell survival (12).

Immune scoring of COVID-19 lung biopsies reveal massive myeloid infiltration, specifically by monocytes, M1 macrophages, and neutrophils (146), which confirms the predominance of these cells in severe COVID-19. Insignificant lymphoid scores for such samples suggest a submerged status of adaptive immunity in severe COVID-19 (147). By using single-cell transcriptomic analysis, Shaath et al. (148) show that bronchoalveolar lavage fluid (BALF) macrophages of patients with severe COVID-19 display pro-inflammatory gene expression signatures; the functional categories include inflammatory response and chemotaxis of myeloid cells, phagocytes, and granulocytes.

Liao et al. (149) used the emerging single-cell RNA sequencing (scRNA-seq) and single-cell TCR sequencing to characterize BALF cells isolated from COVID-19 patients. The results indicate that monocyte-derived macrophages, which replace AMs and predominate among macrophage lineages in the severely damaged lung, are highly inflammatory and potent chemokine producers. Massive clonal expansion of CD8+ T cells in the lungs of patients with mild course of the disease is consistent with the notion on critical importance of rapid and robust adaptive immune response for the control of COVID-19. The leading role of adaptive immunity in COVID-19 is also emphasized in a recent study by Grant (150) demonstrating persistent enrichment of the alveolar space with T cells and monocytes in the majority of patients with SARS-CoV-2 infection.

## The Role of AMs in SARS-CoV-2-Associated ARDS

There is increasing evidence that ARDS pathogenesis depends on macrophages, including resident AMs and transient monocytes/macrophages recruited from the blood (49, 151). AMs and IMs play crucial roles in ARDS, with the timing and degree of macrophage polarization determining severity of the disease and its outcome (152).

High COVID-19 morbidity and mortality in patients with diabetes, chronic obstructive pulmonary disease (COPD), or congestive heart failure (CHF) have been associated with increased counts of AMs in BALF (153). Increased AM counts in BALF have been also observed in patients with COPD, correlating with the disease severity (154), in mice following protracted exposure to diesel exhaust particles (155), in aged rodents (156), in mice subjected to heart failure following augmented hypertension (157), and in models of dilated cardiomyopathy combined with exercise (158). Furthermore, experimental aging and diabetic state was associated with altered distributions of AM phenotypes and their reduced bactericidal capacity (159–161). Accordingly, increased susceptibility to severe COVID-19 can be associated with increased content of AMs that might facilitate homing of COVID-19 by their abundant expression of ACE2.

In severe COVID-19, AMs expand to alveolar cavities in a mixed infiltrate with neutrophils and lymphocytes (147). The SARS-CoV-2-induced aggregation of AMs is evident throughout the acute inflammatory response (147, 162, 163). At the early phase of ARDS resident AMs are highly prone to M1 polarization mediated by activation of TLRs or other pattern recognition receptors (PRRs). Macrophages generally express various types of PRRs and are capable of recognizing viral pathogen-associated molecular patterns (PAMPs) (164, 165). The resulting M1-polarized AMs act as a first line of defense from pathogenic factors including viruses, bacteria, and endotoxins (166–168). M1-polarized AMs release ROS and a variety of pro-inflammatory cytokines including IL-1β, IL-6, IL-18, MCP-1, MIP-2, and TNFα (45, 169, 170). The production of pro-inflammatory cytokines by AMs stimulates immigration of neutrophils from circulation into inter-alveolar septa and alveolar space (39, 171). Administration of natural or synthetic inhibitors of M1 polarization at this stage may allow significant reduction in ALI-associated mortality; attempts to identify and use such substances in ALI/ARDS experimental models have been reported (172–176). Administration of methylprednisolone to mice has been shown to direct AM polarization towards M2 phenotype which helps to preserve oxygenation function of the lungs in ARDS (177). These findings indicate that M1-polarized AMs and their microenvironments play a principal role in ALI/ARDS progression. At the same time, despite their distinct pro-inflammatory behaviors, M1-polarized AMs can express amphiregulin (a potent ligand of the epidermal growth factor receptor) which protects the epithelial barrier, inhibits expression of pro-inflammatory cytokines, and reduces the degree of inflammation; the effect has been demonstrated using a lipopolysaccharide-induced ALI model (178, 179). No data on possible alterations of amphiregulin expression in COVID-19 are available as yet.

A link between COVID-19 severity and repression of the PPARγ complex by AMs, recently demonstrated by Desterke et al. (146), confirms the impact of this macrophage dysregulation in COVID-19 as well as in other viral infections (180–182). In patients with critical manifestation of the disease, the aggregation and activation of AMs may be directly related to the cytokine storm.

In addition to their pro-inflammatory properties, AMs can exert anti-inflammatory action. Depletion of AMs during inflammation also reduces the recruitment of other leukocytes to the lungs (118, 164, 183, 184). During the resolution phase of pulmonary inflammation, AMs debride apoptotic cells by phagocytosis. This efferocytosis process competes with the production of pro-inflammatory cytokines and chemokines through secretion of transforming growth factor beta (TGF-β), prostaglandin E2 (PGE2), and platelet-activating factor (PAF) by AMs (185). The massive infiltration and activation of AMs in COVID-19 may represent a shift from classically activated phenotype (M1) towards alternatively activated phenotype (M2) (147). In ARDS, particularly in the case of antibody-dependent enhancement, such shifts may contribute to inflammatory injuries and fibrosis of respiratory tracts (186). It has been suggested that SARS-CoV-2 can enter AMs *via* the interaction between S protein and ACE2 receptor, given high levels of S protein binding and high receptor density of ACE2 on the surface of LMs (142, 147).

The newly described IM subpopulation of NAMs represents a prospective target for ARDS therapy. These cells express strong regulatory gene profiles and play an important role in regulating pulmonary inflammation *in vivo*. NAMs have been shown to proliferate robustly after influenza infection and activation with the polyinosinic:polycytidylic acid [Poly(I:C)]; in their absence the inflammatory response is augmented, which results in excessive production of pro-inflammatory cytokines and innate immune cell infiltration (120). Viral infection in the absence of NAMs is associated with an excess of pro-inflammatory cytokines and chemokines such as IL-6, CCL2, CCL3, and CCL5, eventually leading to massive lung damage and death (187).

A minor population of intravascular LMs is apparently involved in early stages of ARDS pathogenesis; these cells contribute to vascular injury by excessively aggregating in lung capillaries and producing pro-inflammatory factors in response to PAMPs (121, 188).

Inflammatory reactions in the lungs are invariably supported by immigration of recruited macrophages (21, 22). In ARDS, peripheral blood monocytes accumulate in the lungs to invade pulmonary tissues where they differentiate into M1 macrophages (50, 117).

At later stages of ARDS, M1 macrophages facilitate ECM degradation by producing matrix metalloproteinases (MMPs) and various chemokines, e.g. CXCL10 (189, 190), which support the reversal of fibrosis. M1 macrophages may also induce the expression of MMP-13 and MMP-3 by myofibroblasts (190, 191). M2 macrophages produce anti-inflammatory cytokines and tissue inhibitors of metalloproteinases (TIMPs) which interfere with excessive ECM remodeling and promote its deposition (190, 192). For instance, myofibroblasts can be induced to deposit ECM complexes upon co-cultivation with M2 macrophages (190). The presence of M2 macrophages in pulmonary lesions indicates progression of fibrosis; sustained expression of IL-4, IL-13, TGF-β, and Arg-1 may facilitate collagen deposition (193, 194). The degree of myofibroblast activation and ECM deposition, and accordingly the degree of resulting fibrosis, depend on the balance of M1- and M2-polarized macrophages among other factors of local microenvironment in pulmonary lesions.

Thus, ARDS pathogenesis involves both the recruited pro-inflammatory monocytic macrophages and the anti-inflammatory (or hybrid) resident AMs. Cell therapies involving M2 polarization/reprogramming are highly relevant for the treatment of acute pulmonary inflammation. An enforced shift in macrophage polarization towards anti-inflammatory phenotypes may alleviate ARDS symptoms (49, 195) and save lives.

## Macrophage-Based Cell Therapies for ARDS

There are no 100% effective therapies for ARDS, especially for SARS-CoV-2-associated ARDS; the treatment largely remains symptomatic. Increased survival with ARDS has been achieved over the last decade through the use of mechanical ventilation in moderate and severe cases (196).

In this regard, emerging therapies that can target mechanisms of damage, maintain or enhance defenses of the body against pathogens, and facilitate lung recovery are of great interest. The possibility of regulating macrophage polarization by switching it from M1 to M2 and vice versa (so-called repolarization, or reprogramming) may provide new therapeutic strategies for a wide range of autoimmune and inflammatory diseases including COVID-19 (38, 86, 197–199).

The relevance of research on macrophages for their subsequent use as a target or agent of cell therapy is undeniable. A prospect of regulating cellular homeostasis under pathological conditions by changing the phenotypes of macrophages in damaged tissues and organs, and using reprogramming approaches both *in situ* and *ex vivo* (followed by reverse transplantation of modified macrophages) is most obvious. However, precise mechanisms that govern macrophage polarization remain underexplored. Macrophage polarization is a finely regulated process that involves a wide network of signaling pathways with an abundance of transcription factors and other major regulators. The shifts in M1/M2 balance may occur as a consequence of suppressed M1 polarization or enhanced M2 polarization (or both simultaneously, or vice versa); moreover, some macrophages may remain non-polarized. Enforced M1 polarization of macrophages can stimulate inflammation and cell death, thus providing a useful tool for the treatment of proliferative disorders, notably tumors (200, 201). Enforced M2 polarization of macrophages will have the opposite effect. Increased proportion of M2 macrophages can stimulate regeneration, angiogenesis and ECM remodeling, thus providing a useful tool for the treatment of inflammatory disorders. Obtaining a stable M2-polarized line of macrophages represents a highly relevant goal for transplantology (202).

The number of articles about the ARDS therapy by directed polarization of macrophages towards the desired phenotype is insufficient and all published articles refer to recent years (39, 40, 195, 203–205). The objective of manipulating the phenotype in the exudative phase of the ARDS, when macrophages are predominantly proinflammatory, is an attractive strategy for preventing the manifestations of the acute phase of the disease. Thus, in the work of Wang and colleagues, researchers developed P12 gold nanoparticles coated with hexapeptides with the sequence CLPFFD and investigated their ability to influence the phenotype of alveolar macrophages in LPS-induced mice model of ALI (203). They demonstrated that P12 promoted a significant decrease of M1 cytokines levels (IL-12p40 and IFN-γ) and increase of M2 cytokines levels (IL-10, IL-4, IL-13) in the BALF. *In vitro* experiments showed that P12 nanoparticles reduce the LPS-induced growth in pro-inflammatory cytokines in a culture of bone marrow-derived macrophage. As the main hypotheses for the action of P12 particles coated with hexapeptides, the authors suggest the prevention of excessive activation of macrophages at the early stage of ALI/ARDS by inhibiting TLR4 signaling pathways, as well as reprogramming inflammatory macrophages into anti-inflammatory ones, promoting the repair and arrest of inflammation (203).

Another work investigated the specific type of neutrophil death, neutrophil extracellular traps (NETs), and their relationship with macrophage polarization (204). Neutrophils are the first immune cells to arrive at the site of inflammation. The release of NETs, followed by the ejection of DNA strands to trap pathogens, is characteristic of ALI. Song et al. (204) show that NET levels in ARDS patients correlate positively with pro-inflammatory macrophage markers. Using an LPS-induced mouse model of ALI, the authors demonstrate that the use of NET inhibitors (NE inhibitor and PAD4 inhibitor) reduce the number of neutrophils in BALF and mitigate M1-like macrophage polarization, as indicated by reduced levels of pro-inflammatory markers iNOS, CD11c, and CD54 after experimental administration of NET inhibitors. The M1-polarizing effects of NETs have been also confirmed *in vitro*.

Pro- and anti-inflammatory polarization of macrophages has been associated with alterations in their metabolic status: M1 macrophages produce ATP mainly by glycolysis, while M2 macrophages use aerobic respiration (206). Liu et al. suggested intraperitoneal administration of α-ketoglutarate (α-KG) as the means for reprogramming alveolar macrophages in LPS-induced ALI/ARDS model (205). At first, the authors demonstrated that α-KG induced anti-inflammatory properties *in vitro*: α-KG significantly decreased the expression of IL-1β, IL-6, and TNFα and increased the intracellular ATP level in M1-polarized MH-S cells. In the ALI/ARDS murine model, α-KG inhibited the LPS-induced elevated serum levels of inflammatory cytokines IL-6 and IL-12, as well as the expression of pro-inflammatory genes (IL-1β, IL-6, TNFα) triggered by LPS, while stimulating the expression of M2 marker genes (Arg1 and Mrc1) in the lungs. Taken together, these data indicate that α-KG attenuates the LPS-induced ALI/ARDS by regulating macrophage polarization *in vivo*.

Specifically for ARDS, LMs represent a promising therapeutic target. Effective control over their activation and polarization will help to deter excessive inflammation and limit tissue damage. The potential of targeted macrophage polarization approaches for ARDS treatment was demonstrated in a number of preclinical studies (173–177, 207). On the other hand, unrestrained anti-inflammatory polarization may contribute to fibrosis development and increase susceptibility to secondary infections (208, 209). The articles presented in this section authors did not provide data on the negative effects of the obtained anti-inflammatory polarization of macrophages in LPS-induced ALI/ARDS models, however, such effects should always be taken into account. Probably, the use of transient modification like small noncoding RNAs or chemical agents with controlled factor release is a preferable technology.

One of the most effective methods for obtaining a specific knockout is the use of CRISPR/Cas9 complexes, which can be used to stabilize reprogrammed M1/M2 macrophages. A successful example is provided by knockout of ubiquitin-specific proteinase USP18 involved in IFN signaling in macrophages (210). However, selection of appropriate targets for CRISPR/Cas9 complexes represents a difficult task due to the complexity of metabolic and signaling pathways in macrophages during inflammatory processes. By now, two candidate target genes with differential expression in pro- and anti-inflammatory macrophages have been identified, encoding cytochrome b-245 (*CYBB*) and 7-dehydrocholesterol reductase (*DHCR7*) (211). Modification of these genes may provide macrophages with stable pro- and anti-inflammatory phenotypes as a potential tool for ARDS and COVID-19 cell therapy.

## Conclusion

To summarize, pathogenesis of the new coronavirus infection, especially in cases aggravated by ARDS, depends on phenotypes and functionalities of monocytes, monocyte-derived and resident macrophages in lung tissues. The ratio of pro- and anti-inflammatory macrophages at the sites of injury strongly influences the course and severity of the pathological process in lungs, and this balance represents a potential therapeutic target. The choice of macrophage reprogramming strategy should account for a particular phase of the disease (early or late) in which the treatment is supposed to be administered. For instance, in the early phase of ARDS, resident and recruited AMs are polarized predominantly as pro-inflammatory, consistently with the acute inflammation of lung tissues characteristic of this phase. Therefore, mitigation of this phase would require reprogramming of macrophages towards anti-inflammatory phenotype. By contrast, the next phase of ARDS is marked by massive conversion of both the resident and recruited lung macrophages from pro-inflammatory into anti-inflammatory phenotype. The final phase depends on a fine balance between pro-inflammatory and anti-inflammatory macrophage phenotypes, as both of them are important and special for this phase: pro-inflammatory macrophages facilitate ECM degradation thus limiting fibrosis (preventing excessive fibrosis and ultimately promoting the reversal of fibrosis), whereas anti-inflammatory macrophages express anti-inflammatory cytokines and tissue inhibitors of metalloproteinases thus supporting fibrosis. The degree of ECM deposition and severity of pulmonary fibrosis in this phase strongly depend on the phenotype and functioning of macrophages.

**Figure 2:**
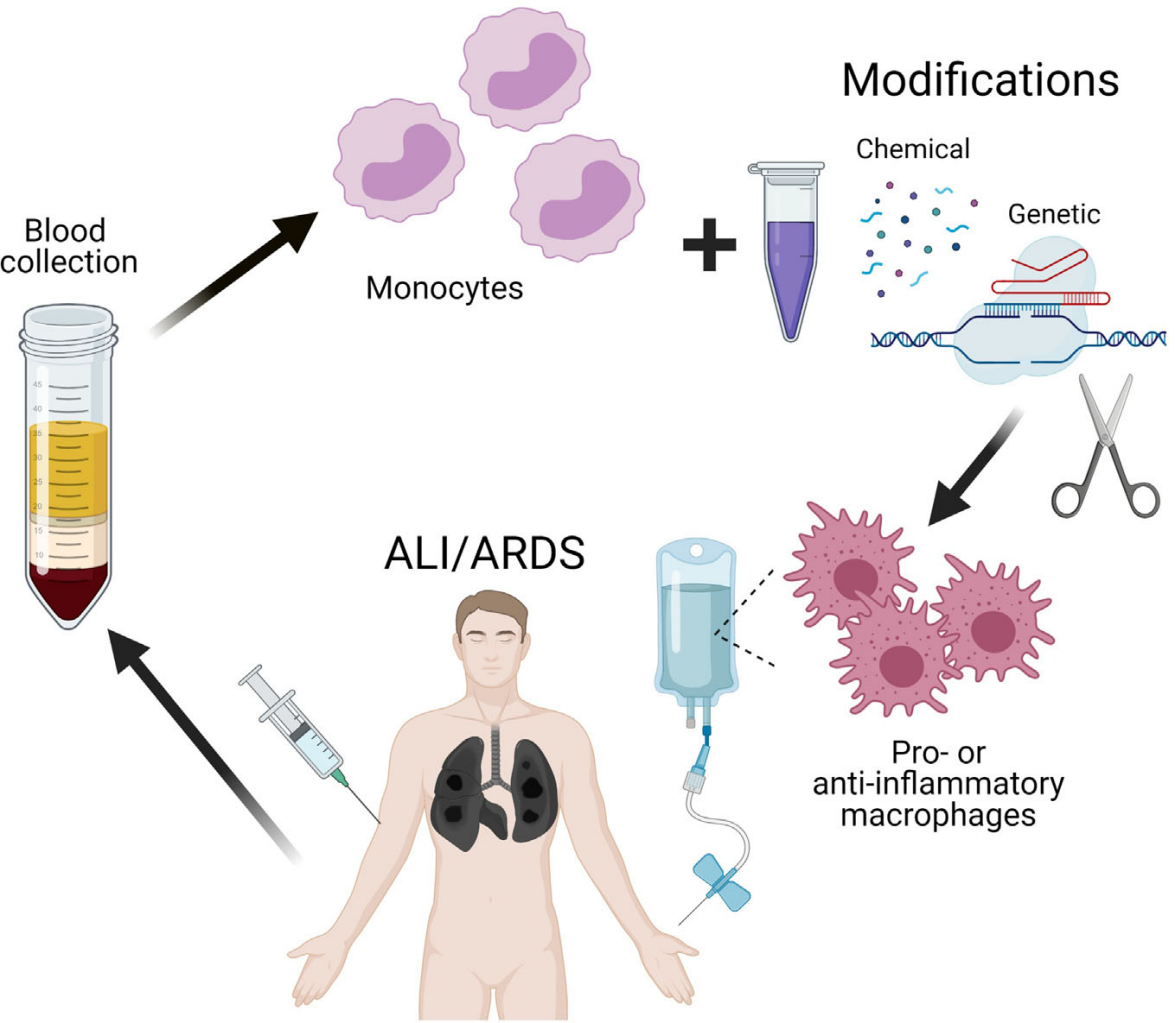
Scheme of perspective therapy of ALI/ARDS by reprogramming of macrophages.

Thus, the use of cell therapy with reprogrammed macrophages for the treatment of ARDS may be successful in COVID-19. Switching macrophage polarization from pro-inflammatory to anti-inflammatory phenotype in the early phase of ARDS can alleviate the enormous production of pro-inflammatory cytokines thereby preventing a cytokine storm and reducing mortality in patients with coronavirus infections. Efficient techniques of macrophage modification (safe and otherwise suitable for clinical purposes) are under way; the approaches include transient genetic modification of macrophages to knock down specific genes responsible for polarization. Convincing preliminary results *in vitro* and *in vivo* were obtained with the use of macrophage cell lines, which are more accessible and easier to modify than primary cultures (212). Continuous improvement of existing methods for delivery of genetic constructs into cells will certainly make it possible to modify primary cultures of macrophages, including isolated blood monocytes, for autologous transplantations (**Figure 2**).

## Author Contributions

AK and TF contributed to conception and design of the research. DD, AL, and PV collected the literature data. DD and AK wrote the first draft of the manuscript. AL and PV wrote sections of the manuscript. All authors discussed and commented the manuscript. All authors contributed to the article and approved the submitted version.

## Funding

This work was supported by the Russian Foundation for Basic Research (grant number 20-04-60027) and the RUDN University Strategic Academic Leadership Program.

## Conflict of Interest

The authors declare that the research was conducted in the absence of any commercial or financial relationships that could be construed as a potential conflict of interest.
